# Clinical course of non-exudative macular neovascularisations in sustained unilateral neovascular age-related macular degeneration

**DOI:** 10.1007/s00417-026-07275-4

**Published:** 2026-05-29

**Authors:** Francesco Cinque, Sofie C. A. ten Brink, Mahfam Shahabi, Anita de Breuk, Thomas. J. Heesterbeek, Caroline C. W. Klaver, Carel B. Hoyng, Yara T. E. Lechanteur

**Affiliations:** 1 Department of Ophthalmology, Donders Institute for Brain, Cognition and Behaviour, Radboud University Medical Center, Geert Grooteplein Zuid 10, 6525 GA Nijmegen, The Netherlands; 2 Department of Ophthalmology, Erasmus Medical Center, Rotterdam, The Netherlands; 3 Department of Epidemiology, Erasmus Medical Center, Rotterdam, The Netherlands; 4 Institute of Molecular and Clinical Ophthalmology, Basel, Switzerland

**Keywords:** Age-related macular degeneration, Retina, Non-exudative macular neovascularisation, Risk

## Abstract

**Purpose:**

To assess the risk of conversion of non-exudative macular neovascularisations (NEMNV) in relation to disease history in sustained unilateral neovascular age-related macular degeneration (AMD).

**Methods:**

This exploratory case-only cohort study included six-monthly visits. Fellow-eye NEMNV were identified using fluorescein and indocyanine green angiography. Patients were stratified by time since first-eye exudation: < 5 years (G1) or ≥ 5 years (G2). The primary outcome was the cumulative annual conversion rate of NEMNV, expressed as conversion rate ratios (CRR) with 95% confidence intervals (CI). Secondary outcomes were independent grading of foveal involvement, core vessel, central core vessel, round shape, sharp borders, and lesion growth.

**Results:**

A total of 134 patients (65.7% female) were included. Mean age at baseline was 74.2 years (SD 8.0). Median time since first-eye exudation was 2.9 years (IQR 1.0–6.3). Patients in G2 had an earlier age at first-eye exudation (66.0 [9.1] years) than G1 (72.7 [7.8]; P < .001). Seven NEMNV lesions were identified (G1:2, G2:5). Cumulative conversion rates were 22.1% overall, 14.3% in G1, and 27.1% in G2. CRR were 2.3 (95% CI 0.6–6.2), 1.3 (95% CI 0.0 – 7.8), and 4.0 (95% CI 0.7 – 16.8). All but one NEMNV showed growth.

**Conclusion:**

In this limited sample, most NEMNV show growth time regardless of conversion to exudative AMD and appear at a higher risk of conversion. The rate of conversion varies with the age of onset of first-eye exudation. Evaluating potential predictive signs of conversion may benefit from incorporating disease history with vascular features.

**Supplementary Information:**

The online version contains supplementary material available at https://doi.org/10.1007/s00417-026-07275-4.

## Key Messages

*****What is known***:**

- Non-exudative macular neovascularisations (NEMNV) are associated with a significantly increased risk of conversion to exudative AMD.
- Existing risk estimates vary widely.
- Proposed predictive factors have focussed primarily on vascular features but do not incorporate age of first-eye exudation.

*****What is new***:**

- Most NEMNV show growth time regardless of whether they convert to exudative AMD.
- The rate of conversion however, may depend on the age of onset of first-eye exudation.
- Predictive assessment may benefit from considering disease history alongside vascular features.

**Supplementary Information:**

The online version contains supplementary material available at https://doi.org/10.1007/s00417-026-07275-4.

## Introduction

Age-related macular degeneration (AMD) is the most common cause of irreversible blindness in the western population [1, 2]. Neovascular, exudative AMD, one of the end stages of AMD, is characterized by exudation and intra- and subretinal fluid accumulation due to a macular neovascularisation (MNV) [3]. The prognosis of exudative AMD has markedly improved with the advent of the anti-vascular endothelial growth factor treatment [4]

Demographically, populations across the world are ageing and since increasing age is the most important risk factor for AMD [5, 6], a surge in cases is expected [1]. Therefore, there is a broad need to accurately assess who is most at risk of developing a sight threatening MNV. Among those most at risk are patients who already developed exudative AMD in one eye, because the risk for progression towards bilateral MNV is relatively high (10% annual) [7].

Evidence suggests that patients with a non-exudative macular neovascularisation (NEMNV) are at a particularly high risk for conversion to exudation [8]. NEMNV, defined as treatment-naïve type 1 neovascularisations without exudation, may be visualized non-invasively using dye based angiography and optical coherence tomography angiography (OCTA) [9]. Other terms that have been used for these NEMNVs include subclinical choroidal neovascularisation and quiescent choroidal neovascularisation.

A study of unilateral neovascular AMD patients, reported that 34.5% of NEMNV became exudative over a period of 2 years, yielding a relative risk of 13.6 compared to non-NEMNV eyes [10]. However, estimates of the risk of annual NEMNV exudation range widely; from 21.1% to 52% [11, 12]. Similarly, the prevalence of NEMNV in unilateral neovascular AMD populations ranges from 6 to 24% [11–17].

This uncertainty regarding timing of conversion hampers clinical management, as doctor’s struggle with scheduling visits and fear the risk of missing early signs of exudation. To address this, we studied the clinical course of NEMNV in the fellow eye of patients with unilateral exudative AMD, specifically focusing on its relationship with the time elapsed since the first eye was diagnosed.

## Method

### Design, setting and participants

Data were collected as part of an ongoing monocentre prospective cohort-study with cross-sectional sampling conducted at the Radboud University Medical Centre in Nijmegen, the Netherlands, aimed at studying progression of AMD in the fellow-eye of patients with unilateral neovascular AMD. The inclusion criteria were unilateral MNV secondary to AMD, while ophthalmic conditions in the fellow-eye that would interfere with the diagnosis and study procedures were grounds for exclusion, including diabetic retinopathy, severe cataract, advanced glaucoma, and retinal vein occlusion. Patients were included from January 2018 to May 2023. Potential participants were identified through ophthalmologists in neighbouring clinics, contact-advertisements in patient association newsletters, and our own clinical population. Patients were followed up for two years or until the fellow-eye developed exudative MNV. If patients discontinued before the two-year mark, they were asked to provide a reason. Where possible, post-study follow-up data were collected either by telephone consultations or review of the electronic patient files. This study was conducted in accordance with the tenets of the Declaration of Helsinki and was approved by the local ethics committee. All provided written informed consent prior to participating.

### Study protocol

At each visit presenting visual acuity (VA) was measured using the Early Treatment Diabetic Retinopathy Study (ETDRS) chart. Demographic and ophthalmic history was assessed through questionnaires. Multimodal imaging was performed in mydriasis.

### Image acquisition

Colour fundus photography was obtained (CFP) (DRI Triton (Topcon corporation, Tokyo, Japan), along with spectral-domain optical coherence tomography (SD-OCT) and optical coherence tomography angiography (OCTA) with 3 × 3 mm, 512 B-scans (Spectralis HRA + OCT (version 6.9.54.0; Heidelberg Engineering, Heidelberg, Germany)) [18, 19]. In the case of a suspected MNV, fluorescein and indocyanine green angiography (FA-ICGA) was performed to confirm its presence and exudation [9].

### Outcomes

The main outcome was the annual cumulative conversion rate for NEMNV. Also, the 95% CI conversion rate ratio (CRR) was calculated. Secondary outcomes were independently graded (yes/no) foveal involvement, core vessel, central core vessel, round shape, sharp borders, growth of NEMNV.

### Stratification

To account for the effect cross-sectional sampling which includes patients at various points in their disease course, all patients were stratified in two groups based on the time between the onset of exudative MNV in the first eye and the study baseline. The difference between onset and study baseline was less than 5 years for group one (G1) and more than 5 years for group two (G2).

### Detection of NEMNV

Detection of NEMNV on OCTA was achieved through screening of several OCTA slabs and thicknesses by (FC) and subsequently reviewed by several retinal specialists (YL, CH), unmasked with open adjudication Screening involved assessment of an *en face* avascular zone slab using the device-specific fully automated segmentation algorithms and projection artifact removal algorithms. Additionally, a custom slab was created by segmenting a layer with Bruch’s membrane as reference with a thickness of 30 microns. Additional settings such as contrast were optimised for appearance of MNV structures. Optimal settings were configured per NEMNV at first appearance and maintained throughout the analysis.

### NEMNV qualitative analysis

Two graders (FC)(YL) independently graded NEMNV according to criteria described by Carnevali, et al*.* at three time points [20]. The following aspects were evaluated: foveal involvement (yes/no), presence of core vessel traveling in the choroid (yes/no), shape of NEMNV (round/irregular), borders (sharp/ill-defined), growth over time (yes/no). Converted NEMNV were graded at first detection, the last available scan before conversion to exudative MNV, and at conversion to exudative MNV. Non-converting NEMNV were graded at first detection, midway between first detection and the last available scan, and at the last available scan. Disagreements were solved by open adjucation.

### Grading

Experienced graders of the Eye-NED Reading Center graded CFP and SD-OCT multimodally for features AMD-features [21]. A modified version of the Wisconsin Age-Related Maculopathy Grading System (WARMGS) [22] was used to grade all AMD features, which were subsequently classified into the Rotterdam Study (RS) classification [21]. SD-OCT scans were graded by FC for presence of complete retinal pigment epithelium and outer retinal atrophy (cRORA) according to criteria by the Classification of Atrophy Meetings group [23].

### Statistics

Baseline characteristics for G1 to G2 were statistically evaluated as follows: categorical data via chi-square tests, continuous data using an independent samples T-test. The annual cumulative conversion for NEMNV per group was calculated by dividing the number of converted NEMNV by the NEMNV person-time to obtain the incidence rate, which was then converted to cumulative conversion using the standard exponential formula under a constant hazard assumption [24]. The conversion rate ratio per group was calculated with formula (1) derived from the incidence rate ratio [25].

1 $$CRR= \frac{{NEMNV}_{1}/{PY}_{1}}{{NEMNV}_{0}/{PY}_{0}}$$

CRR: conversion rate ratio.

NEMNV_1_: no. of converted NEMNV.

PY_1_: person-years NEMNV at risk.

NEMNV_0_: no. of non-NEMNV conversions.

PY_0_: person-years of non-NEMNV at risk.

A 95% CI of the CRR was calculated via Poisson exact methods. (R 4.1.3 (2022–03–10) (R Core Team, Boston, MA, 2014). The *epitools: Epidemiology Tools* (Aragon 2020) and *exactci* (Fay 2010) packages were used. In case the precise moment of exudation was unknown, the date of first anti-vascular endothelial growth factor injection was used. *P* values < 0.05 were considered statistically significant. All other statistical analyses were performed using IBM SPSS Statistics for Windows, version 24 (IBM Corp., Armonk, N.Y., USA).

## Results

### Study cohort

In total, 148 applications were received for the present study of which 134 met the inclusion criteria. The patients who were not eligible were excluded based on the following revised diagnoses: idiopathic choroidal neovascular membrane (7) acquired vitelliform macular degeneration (3), central serous chorioretinopathy (3), myopia (1).

In total N = 134 (female: 88, 65.7%) were included. Median (IQR) time since first eye exudation was 2.9 (1.0–6.3). Age at baseline was 74.2 (8.0). Mean (SD) follow-up in study was 1.7 (1.3) years ranging 0 to 6.0 where 0 reflects eligible patients who developed exudation in the fellow-eye at the screening visit. Fellow-eye grading was as follows; RS stage 0 (31, 23.1%), RS stage 1 (0, 0%), RS stage 2 (44, 32.8%), RS stage 3 (33 (24.6%), cRORA (20, 14.9%). (Table 1).

**Table 1:** Baseline Characteristics

	**Total**
	**(N = 134)**
Time since onset of exudative AMD, median (IQR),	2.9 (1.0–6.3)
Female, No. (%)	88 (65.7%)
Age diagnosis exudative AMD, mean (SD), years	70.0 (8.7)
Baseline age, mean (SD), years	74.2 (8.0)
Lifestyle and ophthalmic history	
Smoking (yes), No. (%)	10 (7.5%)
Ex-smokers (yes), No. (%)	81 (61.2%)
Smoking(ever), No. (%)	92 (68.7%)
Lutein and zeaxanthin supplements (yes), No. (%)	32 (58%)
University level education^*^ (yes), No. (%)	48 (35.8%)
Number of injections, median (IQR)^†^	13 (7–30)
Fellow-eye^‡^	
RS stage 0	31 (23.1%)
RS stage 1	0 (0%)
RS stage 2	44 (32.8%)
RS stage 3	33 (24.6%)
cRORA, No. (%)	20 (14.9%)
Visual acuity	
Visual acuity first eye, mean (SD), letters	58.5 (24.1)
Visual acuity fellow-eye, mean (SD), letters	81.7 (7.6)

NA, not applicable. cRORA, complete retinal pigment epithelium and outer retinal atrophy. RS, Rotterdam Study. RS stage 2: Rotterdam Study stage 2; Soft indistinct drusen (≥ 125 µm) or reticular drusen only, soft distinct drusen (≥ 63 µm). nAMD or neovascular age-related macular degeneration

^*^university refers to having a degree from a practical university (in Dutch: *hoger beroeps onderwijs*) or higher, missing = 8, ^†^missing = 25. ^‡^missing = 4

In total, 32 left the study before the 2-year study period was completed. The reasons for premature discontinuation were: death (8), finding the study too burdensome (7), other non-ophthalmic illnesses (6), travel distance (5), participating in a rival ophthalmic study (2), other (4). The study design is represented in a flowchart (Online Resource Figure 1).

### Group 1 and 2 characteristics

G1 featured 92 patient, G2 featured 42. Age at baseline did not differ for both groups (G1: mean [SD] age 73.8 [7.8], G2: 75.0 [8.3], *P* = 0.4) whereas age of exudative AMD onset did (G1: mean [SD] age 72.7 [7.8], G2: 66.0 [9.1], *P* < 0.001) (Table 2).

**Table 2:** Group 1 and 2 characteristics

	Group 1 Less than 5 years	Group 2 More than 5 years	*P* value
	**(n = 92)**	**(n = 42)**	
Time since onset of exudative AMD, median (IQR), years	1.6 (0.6–3.1)	9.0 (6.4–10.8)	NA
Female, No. (%)	62 (67.4%)	26 (61.9%)	0.5
Age at onset exudative AMD, mean (SD), years	72.2 (7.8)	66.0 (9.1)	< 0.001*
Baseline age, mean (SD), years	73.8 (7.8)	75.0 (8.3)	0.4
Lifestyle and ophthalmic history			
Smoking (yes), No. (%)	8 (9.8%)	1 (2.4%)	0.1
Ex-smokers (yes), No. (%)	49 (53.3%)	33 (78.6%)	0.01*
Smoking(ever), No. (%)	58 (63.0%)	34 (81.0%)	0.04*
Lutein and zeaxanthin supplements (yes), No. (%)	55 (59.8%)	22 (52.4%)	0.4
University level education^*^ (yes), No. (%)	33 (35.9%)	15 (35.7%)	0.8
Number of injections, median (IQR)^†^	11 (6–20)	32 (14–51)	0.01*
Fellow-eye ^‡^			
RS stage 0	21 (22.8%)	10 (23.8%)	0.9
RS stage 1	0 (0%)	0 (0%)	NA
RS stage 2	37 (40.2%)	7 (16.7%)	0.01*
RS stage 3	20 (21.7%)	13 (31.0%)	0.3
cRORA, No. (%)	9 (9.8%)	11 (26.2%)	0.01*
Visual acuity			
Visual acuity first eye, mean (SD), letters	65.1 (19.5)	44.6 (26.8)	< 0.001*
Visual acuity fellow-eye, mean (SD), letters	82.4 (5.6)	80.3 (10.3)	0.1

NA, not applicable. cRORA, complete retinal pigment epithelium and outer retinal atrophy. RS, Rotterdam Study. RS stage 2: Rotterdam Study stage 2; Soft indistinct drusen (≥ 125 µm) or reticular drusen only, soft distinct drusen (≥ 63 µm). nAMD or neovascular age-related macular degeneration

*Indicates *P* < 0.05. ^*^university refers to having a degree from a practical university (in Dutch: *hoger beroeps onderwijs*) or higher, missing = 4,4. ^†^missing = 13, 12. ^‡^missing = 4,0

G2 contained more ex-smokers (G1: 49 (53.3%), G2: 22 (78.6%), *P* = 0.01) and ever-smokers than G1 (G1: 58 (63.0%), G2: 34 (81.0%), *P* = 0.04). Other lifestyle factors were similar across groups. G2 had the highest proportion of cRORA (G1: 9 (9.8%), G2: 11 (26.2%), *P* = 0.01) and the lowest proportion with RS stage 2 (G1:37 (40.2%), G2: 7 (16.7%), *P* = 0.01). G2 had worse visual acuity in the first eye (G1: mean [SD] letters 65.1 [19.5], G2: 44.6 [26.8], *P* < 0.001) while mean visual acuity for the fellow-eye did not differ (Table 2).

### Main results

Seven NEMNV were detected, of which 2 were in G1. Notably, 2 NEMNV were initially missed and were detected during follow-up. However, all NEMNVs were present at baseline after in-depth examination of images. No incident NEMV were detected during follow-up. Four out of the 7 NEMNV became exudative during follow-up with a median conversion time of 1.5 ranging from 10 months to 3 years. For the total group (N = 134) annual cumulative conversion of NEMNV was 22.1% with a CRR of 2.3 (95% CI 0.6–6.2). (Table 3) In G1, annual cumulative conversion of NEMNV was 14.3% with a CRR of 1.3 (95% CI 0.0–7.8) while in G2 this was 27.1% with a CRR 4.0 (95% CI 0.7–16.8).

**Table 3:** NEMNV and conversion rate ratio

	Total (N = 134)		G1: Less than 5 years (n = 92)		G2: More than 5 years (n = 42)	
	NEMNV	No NEMNV	NEMNV	No NEMNV	NEMNV	No NEMNV
Converted	4	31	1	23	3	8
Person-years	16.0	290.1	6.5	188.2	9.5	101.9

Abbreviations: NEMNV, non-exudative macular neovascularisation; CRR, conversion rate ratio; person-years, the total amount of time each patient was at risk of conversion, summed

### Descriptive and qualitative analysis of NEMNVs

**Fig. 1:**
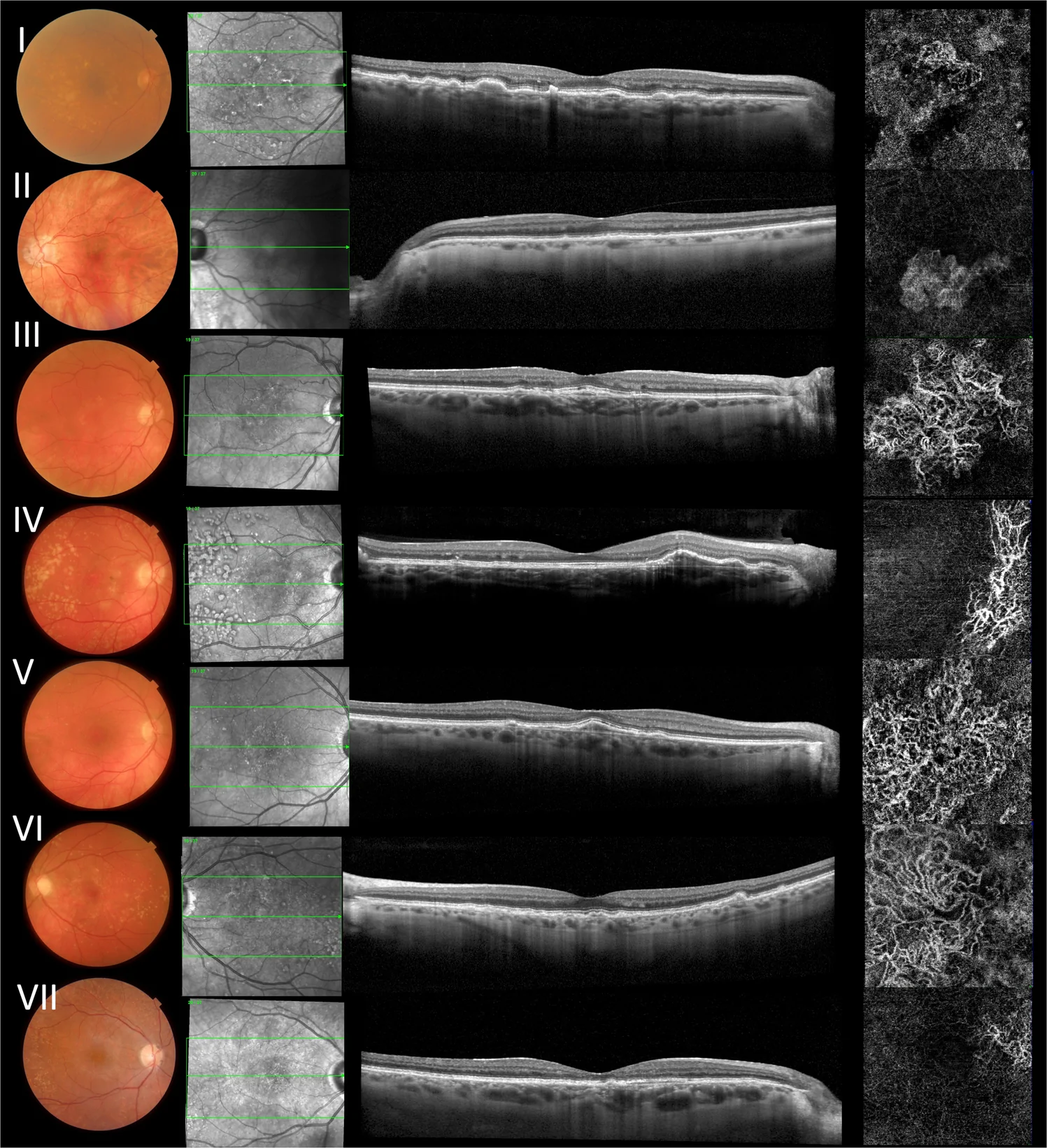
Baseline Imaging of Non-exudative Macular Neovascularisations

**Fig. 2:**
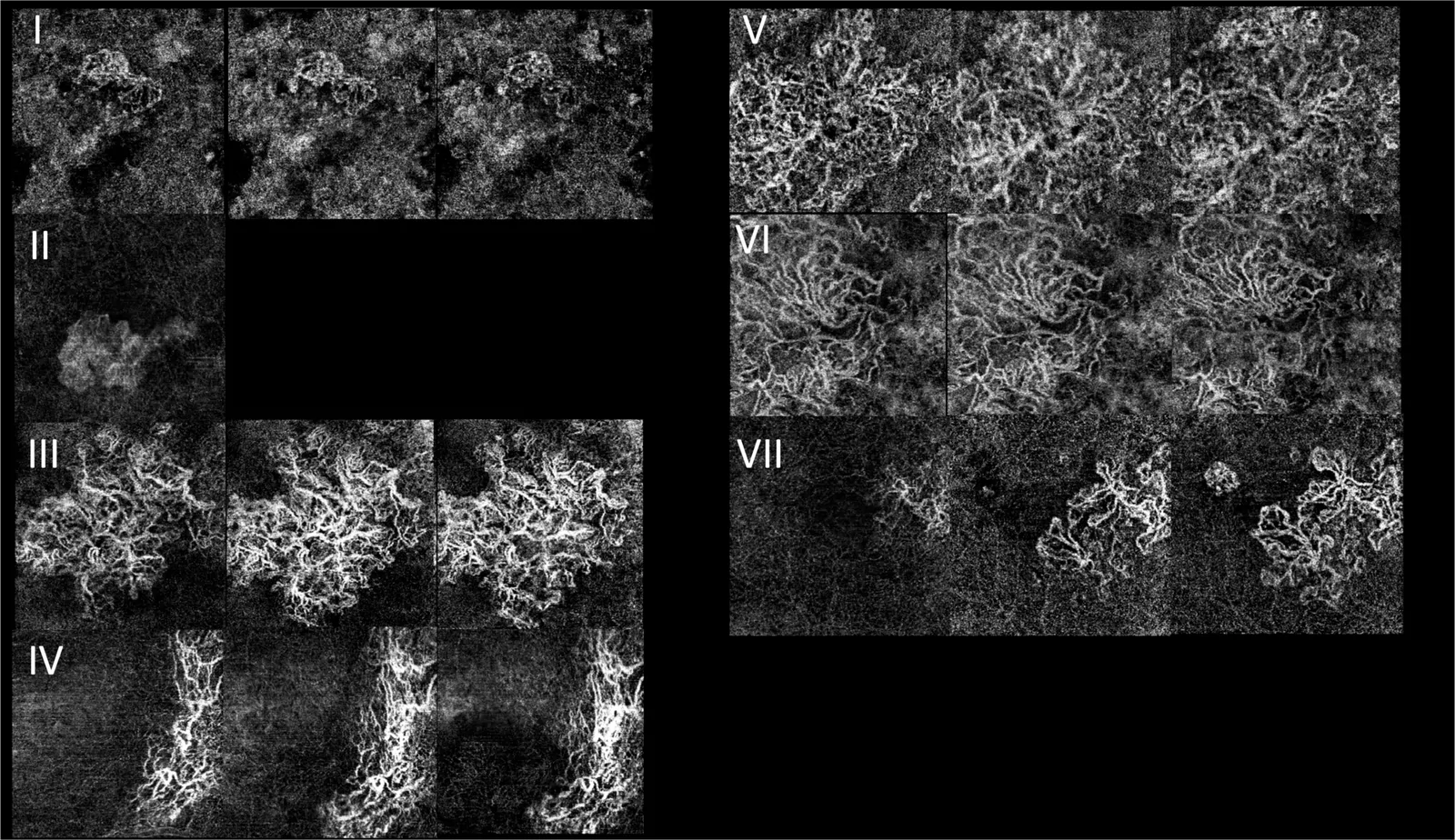
OCTA of Non-exudative Macular Neovascularisations at Different Visits

All NEMNV patients were female, the median age at baseline was 72 and ranged from 43 to 92 years. All NEMNV-eyes showed drusen and none showed cRORA. Three of 7 were graded as RS stage 3, the others were graded as RS stage 2. All NEMNVs displayed sharply defined borders at baseline (Fig. 1). We identified a core vessel in all cases, whereas a central core vessel was visible in 3 cases. Three NEMNV displayed foveal involvement. Only one NEMNV was shaped roundly. For growth analysis, 6 out of 7 had available follow up. Of these NEMNV, 5/6 (83%) displayed growth between baseline and visit 2. Of the NEMNV that became exudative (patient I-IV) all (100%) showed growth. Non-converting NEMNV showed growth in most cases (2 of 3 or 66%). (Fig. 2) Between visit 2 and 3, 3 NEMNV showed growth (Online Resource Table 1).

Baseline imaging of all non-exudative macular neovascularisations (NEMNV) for patient I-VII, right to left panel: colour fundus photography (CFP), infra-red image, spectral domain optical coherence tomography (SD-OCT), optical coherence angiography (OCTA). I. Female, 78 years, settings: Bruch’s membrane with depth of 40 micron. II. female, age 92 years, settings: avascular zone. III. Female, age 74 years, settings: artifact removal, contrast 1:5, retinal pigment epithelium to Bruch’s membrane. IV. Female, age 43 years, settings: avascular zone. V. Female, age 72 years, settings: Bruch’s membrane with a thickness of 40 micron. VI. Female, age 67 years, settings: projection artifact removal, contrast 1:7, reference retinal pigment epithelium with a depth of −27 micron and thickness of 40 micron. VII. Female, age 70, settings: avascular zone.

Optical coherence angiography (OCTA) of non-exudative macular neovascularizations (NEMNV) in patients I-VII was analyzed. Patients I-IV developed exudative age-related macular degeneration (AMD). The left panels represent images baseline, the middle panels show the penultimate OCTA images, and the right panels display the OCTA images at the moment of exudation. For patients V-VII, the left panels represent images at diagnosis, the middle panels show images from midway visits, and the right panels display baseline from the last visit. The settings for all images are the same and are detailed below.

- Patient I: A sharply defined, non-foveal lesion with a core vessel showed growth over time between the first and penultimate visits. The time between the first and middle panels is 7 months, and 4 months between the middle and last panels.
- Patient II: A sharply defined, non-foveal lesion with a core vessel was observed. Growth could not be determined due to unavailable images.
- Patient III: A sharply defined, foveal lesion with a central core vessel showed growth between all visits. The time between the first and middle panels is 2.8 years, and 2 months between the middle and last panels.
- Patient IV: A sharply defined, non-foveal lesion with a central core vessel showed growth between the first and penultimate visits. The time between images is 10 months from the first to middle panel and 2 weeks from the middle to last panel.
- Patient V: A sharply defined, foveal lesion with a central core vessel showed growth across visits. The time between images is 1 year 3 months from the first to middle panel and 1 year 1 month from the middle to last panel.

## Discussion

In this exploratory case-only cohort study of N = 134 patients with unilateral exudative AMD, annual conversion of 7 NEMNV was 22.1% with a CRR of 2.3 (95% CI 0.6–6.2). However, risk of conversion varied with time since onset of exudative AMD in the first eye. The annual conversion rate was 27.1% with a CRR of 4.0 (95% CI 0.7–16.8) for patients with more than five years since first eye onset of exudative AMD (G2). In contrast, these figures were 14.3% with a CRR of 1.3 (95% CI 0.0–7.8) for patients whose first eye onset of exudative AMD was less than five years before baseline (G1), This discrepancy raises two important questions: First, how might we explain this contrast and, second, what is the actual risk of conversion for NEMNV?

Baseline differences alone are unlikely to fully account for the apparent contrast. Group 1 and 2 appeared to differ for baseline characteristics following stratification. Plausibly reflecting longer disease duration, G2 featured more severe phenotypes in the fellow-eye and poorer visual acuity in the first eye while lifestyle factors were comparable. In addition, G2 might be more genetically burdened on account of the younger age of onset [26, 27], (Table 2).

Potentially more impactful is that through length-biased sampling overinclusion of patients with longer disease histories will likely have occurred. The resulting sample is left truncated, meaning that patients who have developed exudative AMD in the fellow-eye shortly after first eye exudation were less likely to be included. See conceptual diagram (Online resource Figure 2). Therefore, G2 enrichment with long standing NEMNV may have artificially inflated conversion rate. Biologically, since G2 reflects the more stable disease population (median (IQR) time since first eye exudation was 9.0 (6.4–10.8) years), it seems plausible given the data to suggest that these NEMNV may have had more time to mature only reaching critical risk for exudation once under study. It might therefore be the case that temporal disease context is an important modifier for NEMNV behaviour.

Qualitative analysis shows that most NEMNV show change over time regardless of conversion (e.g. 83% of NEMNV with available follow-up showed growth) (Online Resource Table 1). Growth thus appears to be a common feature but lacks specificity for predicting conversion. To fully appreciate potential predictive signs therefore, future analysis may benefit from analysing potential predictive vascular changes in conjunction with duration of disease history.

This hypothesis has important consequences for the appraisal of current literature. Most if not all similar studies on the natural history or predictive factors of NEMNV make no mention of the effect of length-biased sampling [11–13, 15, 16, 20]. As stated, estimates of the prevalence of NEMNV in AMD populations range from 6 to 24% [11–16]. While existing studies vary in regards to sample size, fellow-eye inclusion criteria, definition and detection method of NEMNV, oversampling patients with longer disease durations (with possibly longer existing NEMNV) could also impact prevalence estimates.

Considerations for future studies include the non-standard imaging protocols and imaging devices [28]. As OCTA devices improve and become more widespread, NEMNV conversion risk may decrease due to earlier detection. Similarly, in the absence of a gold standard for OCTA-based NEMNV detection, convergence of detection methods might similarly improve inter-study validity. This is relevant since in the present study 2 NEMNV were erroneously detected at baseline highlighting the effect of a learning curve. In sum, future studies seeking to improve understanding potential predictive signs of NEMNV exudation may benefit from following NEMNV from the moment of first eye exudation or using an otherwise specified, standardised starting point. This would allow for time to event analyses with which other known risk factors for AMD could be statistically evaluated.

### Strengths and limitations

A major strength of this study is the verification of NEMNV with ICG in combination with the systematic multimodal visits. This study is primarily limited by the small number of NEMV (7) and events (4). Stratification on time since first eye exudation further results in lower power with subsequent high uncertainty which is reflected in the wide 95% CI of CRR’s. However, stratification is necessary to curtail survivor bias which is a limitation of the present design on account of length-biased sampling (Online resource Figure 2).

## Supplementary Information

Below is the link to the electronic supplementary material.

Supplementary file1 (PDF 94 KB)

Supplementary file2 (PDF 10 KB)

Supplementary file3 (DOCX 14 KB)

## Data Availability

No datasets were generated or analysed during the current study.
