# Supplementary material for: Clinical course of non-exudative macular neovascularisations in sustained unilateral neovascular age-related macular degeneration
Source: Graefes Arch Clin Exp Ophthalmol. 2026 May 29;264(11):3707–15. doi: 10.1007/s00417-026-07275-4 (PMC13736464; doi:10.1007/s00417-026-07275-4)
Supplement: Supplementary file 1 [file 417_2026_7275_MOESM1_ESM.pdf]

Supplemental Figure 1. Flow-chart of Study

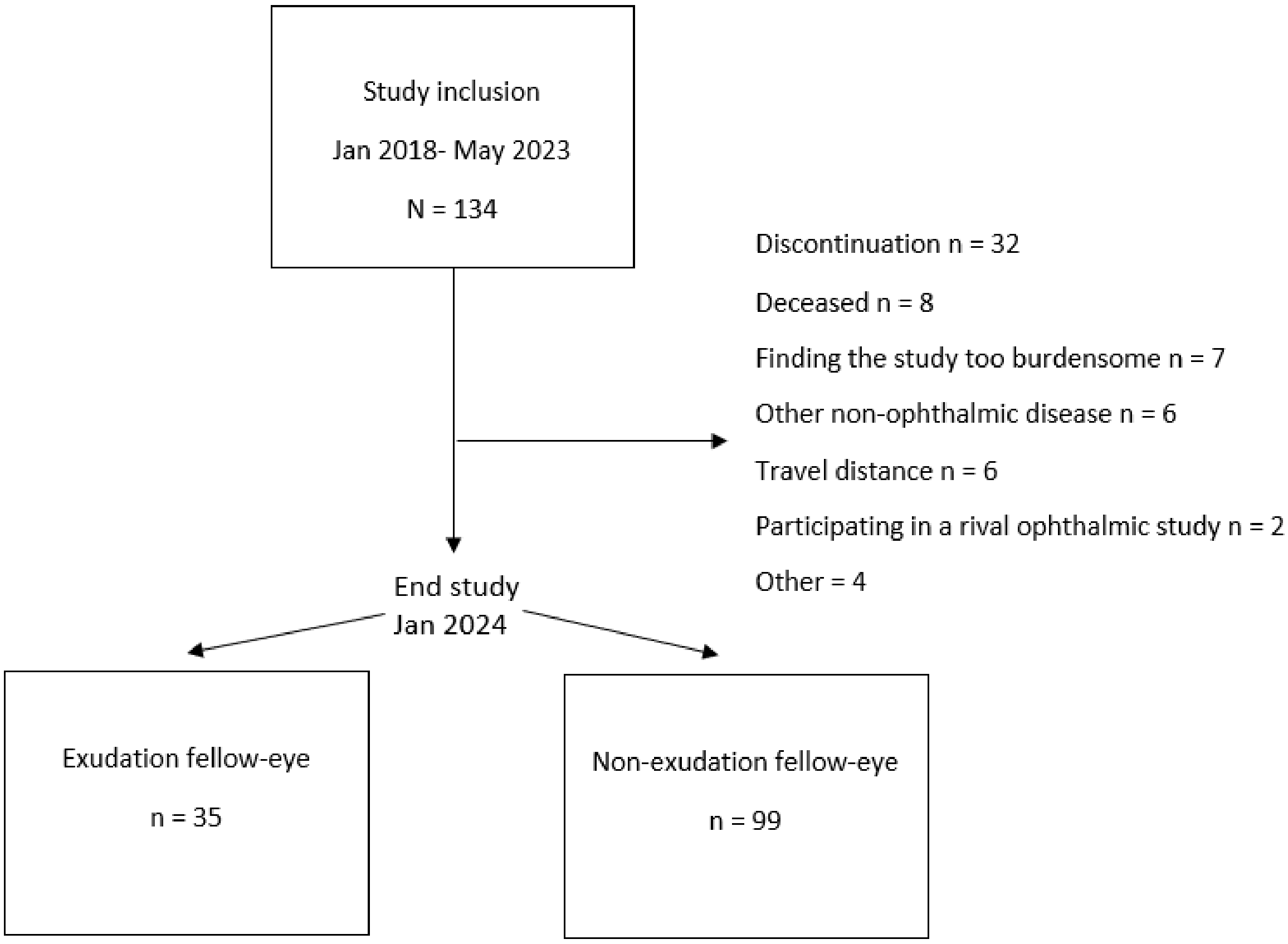

Supplemental Figure 1 shows a flow-chart of this study and its main events.
