# Supplementary material for: Clinical course of non-exudative macular neovascularisations in sustained unilateral neovascular age-related macular degeneration
Source: Graefes Arch Clin Exp Ophthalmol. 2026 May 29;264(11):3707–15. doi: 10.1007/s00417-026-07275-4 (PMC13736464; doi:10.1007/s00417-026-07275-4)
Supplement: Supplementary file 2 [file 417_2026_7275_MOESM2_ESM.pdf]

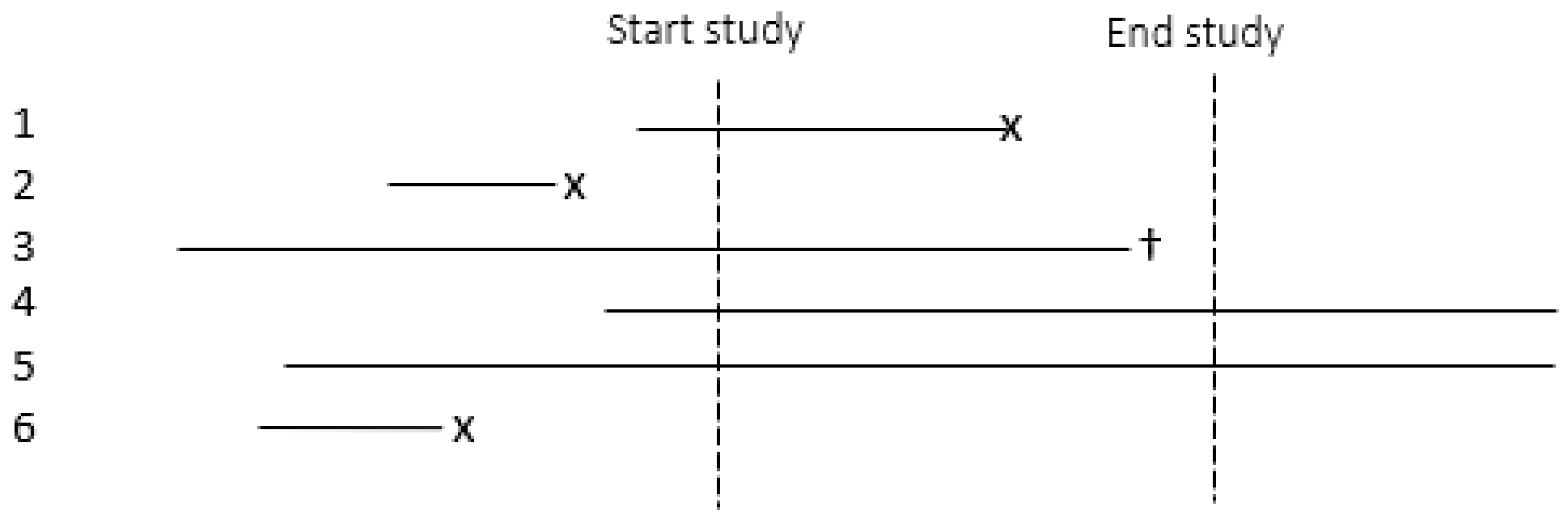

x: nAMD in the fellow eye
†: patient has died
