# Supplementary material for: Clinical course of non-exudative macular neovascularisations in sustained unilateral neovascular age-related macular degeneration
Source: Graefes Arch Clin Exp Ophthalmol. 2026 May 29;264(11):3707–15. doi: 10.1007/s00417-026-07275-4 (PMC13736464; doi:10.1007/s00417-026-07275-4)
Supplement: Supplementary file 3 [file 417_2026_7275_MOESM3_ESM.docx]

**Online Resource Table 1. Qualitative analysis NEMNV**

| Patient | I (G2) | II (G2) | III (G1) | IV (G2) | V (G2) | VI (G1) | VII (G2) |
| --- | --- | --- | --- | --- | --- | --- | --- |
| Foveal involvement | No | No | Yes | No | Yes | Yes | No |
| Core vessel | Yes | Yes | Yes | Yes | Yes | Yes | Yes |
| Central core vessel | No | No | Yes | Yes | Yes | Unclear | No |
| Round shape | No | No | No | No | Yes | Unclear | Unclear |
| Sharp borders | Yes | Yes | Yes | Yes | Yes | Yes | Yes |
| Growth visit 1-2 | Yes | NA | Yes | Yes | Yes | No | Yes |
| Growth visit 2-3 | No | NA | Yes | No | Yes | No | Yes |

Abbreviations: NEMNV, non-exudative macular neovascularisation. 3 Supplemental table 1 shows the grading results of qualitive analysis of NEMNV.
